# Current New Approach in Thoracoscopic Surgery: Non-Intubated Uniportal Video-Assisted Thoracoscopic Surgery (NI-UniVATS)

**DOI:** 10.3390/medicina61040641

**Published:** 2025-04-01

**Authors:** Mehmet Agar, Ilham Gulcek, Muhammed Kalkan, Hakki Ulutas, Muhammet Reha Celık, Ahmet Aksu, Siyami Aydın, Muharrem Cakmak

**Affiliations:** 1 Department of Thoracic Surgery, Medicine Faculty, Firat University, 23200 Elazig, Turkey; siyamiaydin51@gmail.com (S.A.); g.c.dr.ckmk@gmail.com (M.C.); 2 Department of Thoracic Surgery, Gaziantep City Hospital, 27470 Gaziantep, Turkey; ilhamgulcek@gmail.com; 3 Department of Thoracic Surgery, Malatya Training and Research Hospital, 44330 Malatya, Turkey; muhammedkalkan1993@gmail.com; 4 Department of Thoracic Surgery, Medicine Faculty, Izmir Economics University, 35330 Izmir, Turkey; drhakkiulutas@gmail.com; 5 Department of Thoracic Surgery, Medicine Faculty, Atilim University, 06805 Ankara, Turkey; rehacelik@gmail.com; 6 Department of Anaesthesiology and Reanimation, Faculty of Medicine, Firat University, 23200 Elazig, Turkey; drahmetaksu@gmail.com

**Keywords:** NI-UniVATS, non-intubated, minimally invasive, uniportal, video-assisted thoracoscopic surgery

## Abstract

*Background and Objectives:* Non-intubated uniportal video-assisted thoracoscopic surgery (NI-UniVATS) is a minimally invasive technique performed using a single port, allowing the entire surgical procedure to be completed with spontaneous breathing without the need for general anesthesia. *Materials and Methods:* This retrospective study included 51 patients who underwent NI-UniVATS between 2020 and 2023. The intraoperative and postoperative data of patients who underwent NI-UniVATS were evaluated. *Results:* Among the cases, 37 (72.5%) were male, and 14 (46.6%) were female, with a mean age of 47.73 ± 20.43 years (range: 18–78 years). The mean operative time was 25.92 ± 7.31 min. No perioperative complications were observed in any patient. The mean postoperative hospital stay was 4.17 ± 1.76 days (range: 2–9 days). A right hemithoracic approach was performed in 28 patients (54.9%), whereas a left hemithoracic approach was used in 23 patients (45.1%). The procedures performed included wedge resection in 27 patients (52.9%), biopsy in 22 patients (43.1%), pericardial window creation in one patient (2%), and intrathoracic foreign body removal in one patient (2%). *Conclusions:* NI-UniVATS allows for safer surgery by preventing the adverse effects and complications associated with general anesthesia. NI-UniVATS can be recommended as a safe and feasible approach for both minor and major thoracic procedures.

## 1. Introduction

Video-assisted thoracoscopic surgery (VATS) is a minimally invasive approach that has introduced a new paradigm in thoracic surgery, emerging as a strong alternative to traditional open surgery. Simultaneously, scientific advancements in anesthesia and surgical techniques have facilitated a shift from aggressive methods to minimally invasive approaches for the treatment of pleural, mediastinal, and pulmonary diseases. Although VATS has demonstrated several advantages over conventional open surgery, it still requires general anesthesia and endotracheal intubation. However, in recent years, non-intubated uniportal video-assisted thoracoscopic surgery (NI-UniVATS), a less invasive approach that does not require intubation, has emerged as a prominent new technique in thoracic surgery. In our study, we aim to share our clinical outcomes related to this evolving technique and contribute to the literature for future research.

NI-UniVATS represents an advanced version of modern video-assisted thoracoscopic surgery (VATS). It refers to video-assisted thoracoscopic surgery performed using a single port and differs significantly from the conventional VATS technique. NI-UniVATS allows the entire surgical procedure to be completed with spontaneous breathing without the need for general anesthesia [1]. In this technique, surgery is performed through a single-port entry with minimal sedation and local anesthesia, thereby enhancing surgical efficiency while minimizing the adverse effects of anesthesia on patients.

Uni-VATS was first performed by Jacobaeus in 1910 [2]. With advancements in anesthesia, intraoperative oxygenation, imaging techniques, and surgical instruments, VATS became a widely used method for both diagnosis and treatment in the 1990s [3]. Over time, the technique has evolved further, leading to the development of awake or non-intubated Uni-VATS.

This study aims to analyze the clinical and operative data of patients who underwent NI-UniVATS, evaluating the advantages, disadvantages, and challenges associated with the procedure. The primary objective is to determine how NI-UniVATS improves clinical outcomes compared to conventional methods, identify which stages of the surgical process benefit from this technique, and demonstrate its practicality and applicability for lung pathologies.

## 2. Materials and Methods

### 2.1. Patients

A total of 51 patients who underwent NI-UniVATS between 2020 and 2023 were retrospectively evaluated. All patients were analyzed in terms of gender, age, surgical side, performed surgical procedure, comorbid conditions, complications, operative time, length of hospital stay, and Visual Analog Scale (VAS) scores. This research was conducted in accordance with the Ethics Committee of the Firat University Scientific Research and Publication Ethics Board (2024/11-17) (approval date: 1 August 2024). The study was carried out following the ethical standards of the 1964 Declaration of Helsinki.

### 2.2. Statistical Analysis

Data analysis was performed using IBM^®^ SPSS^®^ Statistics (version 25 for Windows, IBM Corporation, Armonk, NY, USA). Normality analysis was assessed using the Shapiro–Wilk test, histogram distribution, and skewness-kurtosis parameters. Descriptive statistics were presented as mean ± standard deviation for normally distributed variables, median (min-max) for non-normally distributed variables, and frequency (percentage) for nominal variables. A *p*-value of <0.05 was considered statistically significant.

### 2.3. Anesthesia Technique

For non-intubated patients, following appropriate cannulation and monitoring, continuous oxygen was administered via a nasal cannula or face mask at a flow rate of 4–6 L/min throughout the operation. Immediately before the surgical incision, an intravenous bolus of propofol (1–2 mg/kg) was administered. To maintain mild sedation, intermittent bolus doses of 20–30 mg propofol were given, not exceeding a total dose of 200 mg. Additional doses were administered intermittently in cases of inadequate sedation, indicated by increased heart rate, respiratory rate, or patient movement.

### 2.4. Surgical Technique

Patients were positioned in a thoracotomy position with the surgical site facing upwards. Local anesthesia was administered using 2% lidocaine at thefifth intercostal space—either anterior, mid, or posterior axillary line—based on the localization of the lesion. A 3–4 cm utility incision was made at the planned surgical site. After dissecting the anatomical structures, the intrathoracic space was accessed. A video thoracoscope was inserted at an appropriate angle for exposure through one side of the utility incision and was secured in place using a sterile suture. Surgical instruments were introduced through the utility incision to perform the intrathoracic procedures. Upon completion of the procedure, a chest drain was placed along the posterior margin of the utility incision, secured by compressing it against the muscular structures. Finally, the anatomical layers were closed, and the surgery was completed.

## 3. Results

Among the cases, 37 (72.5%) were male, and 14 (46.6%) were female, with a mean age of 47.73 ± 20.43 years (range: 18–78 years). All patients included in the study underwent NI-UniVATS. A right hemithoracic approach was performed in 28 patients (54.9%), while a left hemithoracic approach was used in 23 patients (45.1%). The mean operative time was 25.92 ± 7.31 min. The mean postoperative hospital stay was 4.17 ± 1.76 days (range: 2–9 days). The perioperative numerical data of the patients according to their clinical diagnoses are presented in Table 1.

Among the operated patients, comorbid conditions included heart failure in five patients, chronic obstructive pulmonary disease in four patients, diabetes mellitus in three patients, obesity in two patients, and chronic kidney disease in one patient. During the procedure, cough was observed in three patients, while one patient exhibited mild panic attack symptoms. No major complications were encountered during the procedure (Table 2).

Postoperative pain scores measured using the Visual Analog Scale (VAS) at different time points were as follows: VAS1 (first postoperative hour): 5.80 ± 1.35, VAS2 (sixth postoperative hour): 3.33 ± 0.65, and VAS3 (24th postoperative hour): 1.74 ± 0.56. Postoperative VAS scores according to the clinical diagnoses of the patients are shown in Table 3.

The surgical procedures performed included wedge resection in 27 patients (52.9%), biopsy in 22 patients (43.1%), pericardial window creation in one patient (2%), and intrathoracic foreign body removal in one patient (2%). The perioperative characteristics of the patients are presented in Table 4

Among the patients, 27 (52.9%) underwent pleural and mediastinal biopsy, while 22 (43.1%) underwent wedge resection. Additionally, a pericardial window was created in one patient, and an intrathoracic foreign body was removed in another. Following the procedures, the most frequently observed pathological diagnosis was bullae, followed by fibrous pleuritis, metastasis, and mesothelioma. Postoperative pathological diagnoses based on the surgical procedures performed and the collected specimens are presented in Table 4.

When comparing patients operated on for pneumothorax and pleural effusion, no statistically significant difference was found in VAS scores or length of hospital stay. However, there was a statistically significant difference in age (*p* < 0.001) and operative time (*p* = 0.005) (Table 5).

Upon further comparison of these two groups, it was observed that NI-UniVATS was preferred for pneumothorax in younger patients and for pleural procedures in older patients. Additionally, wedge resection was the preferred procedure for pneumothorax, whereas biopsy was favored for pleural conditions. Regarding operative time, the pneumothorax group had shorter surgical durations compared to the pleural intervention group.

## 4. Discussion

Initially, multi-port approaches were preferred in thoracoscopic surgery; however, with increasing experience, the number of ports has decreased, leading to the adoption of single-port techniques [3]. Gaetano Rocco revitalized Jacobaeus’ single-port approach for thoracic surgical procedures, performing interventions such as pneumothorax management, biopsy, and wedge resection using a uniportal approach, thus popularizing the technique [4,5]. The combination of minimally invasive techniques and non-intubated anesthesia protocols has been shown to yield highly favorable outcomes for patients [6]. While NI-VATS was initially performed only for limited indications, its applications have expanded to include malignant pleural effusion management, empyema surgery, lobectomy, segmentectomy, bronchial sleeve procedures, and tracheal resections [7,8,9]. In our study, NI-UniVATS was performed on patients with a variety of indications. This technique was applied to patients undergoing treatment for pneumothorax, pleural effusion management, pulmonary nodule and mediastinal lesion evaluation, and pericardial window creation. Our findings demonstrate that a wide range of patient groups can undergo surgery with a uniportal and non-intubated approach, independent of conventional VATS techniques.

The current literature includes numerous recent studies demonstrating that NI-VATS can be safely utilized in the diagnosis and treatment of various surgical diseases (Table 6).

One of the most significant advantages of the NI-VATS technique is the reduction or prevention of complications associated with general anesthesia. In single-lung ventilation, complications related to general anesthesia management include tracheobronchial injury, postoperative nausea and vomiting, sore throat, bleeding, malpositioning of the double-lumen tube, barotrauma, and postoperative respiratory lung complications [19,20,21]. The difficulty in placing the double-lumen tube can lead to minor complications such as sore throat and hoarseness, as well as severe airway complications, including arytenoid dislocation and rupture [22,23]. In a study involving 65 patients, single-lung ventilation was found to contribute to acute lung injury during the perioperative period and induce ischemia-reperfusion injury in the non-ventilated lung [24]. Another study concluded that single-lung ventilation alters the pulmonary microbiome following thoracoscopic surgery [25]. In our study, none of these complications were observed, as patients received only oxygen-supported sedation instead of general anesthesia.

During NI-VATS, the development of iatrogenic pneumothorax can lead to hypoventilation and decreased pulmonary perfusion, potentially resulting in hypercapnia [26]. Additionally, uncontrolled pulmonary artery bleeding during NI-VATS may necessitate emergency thoracotomy [27]. In non-intubated patients, cough reflex, hypercapnia, acidosis, and panic attacks may also be encountered [28]. However, in our study, only three patients developed a cough reflex during surgery, and one patient experienced a mild panic attack. Despite these occurrences, the surgical procedure was successfully and safely completed in all cases.

Although some contraindications for non-intubated surgery have been described in the literature, there is no established consensus on this matter [29]. Certain conditions, such as obesity, excessive movement of the diaphragm or mediastinum, and coughing, may pose challenges to performing NI-VATS, but they are not considered absolute contraindications. In our study, coughing was observed in two cases during NI-UniVATS performed for pneumothorax and in one case undergoing wedge resection for a pulmonary nodule. Additionally, one patient experienced anxiety-related discomfort due to a mild panic attack. However, despite these challenges, all procedures were successfully and safely completed.

In the past, the American Society of Anesthesiologists (ASA) recommended non-intubated techniques only for patients classified as ASA 1–2, with a body mass index (BMI) below 30, cardiopulmonary stability, and no expected difficulty in intubation. However, non-intubated techniques are now being safely used even in high-risk patients [30,31,32]. A study suggested that non-intubated techniques should be preferred in patients with comorbidities such as chronic obstructive pulmonary disease (COPD), who may require intensive care in the postoperative period [33]. Consistent with the literature, in our study, patients with comorbidities such as heart failure, COPD, diabetes mellitus, obesity, and chronic kidney disease successfully underwent NI-UniVATS without experiencing any major complications.

In the literature, the reported conversion rate from non-intubated to intubated surgery ranges from 0% to 11% [34]. In a study by Chen et al., three out of thirty NI-VATS cases performed under thoracic epidural anesthesia required conversion to intubation [35]. In our study, none of the cases required conversion to intubation, demonstrating the feasibility and safety of the NI-UniVATS technique.

In a study conducted by Ahn et al., patients who underwent VATS under intubated and non-intubated conditions were compared in terms of anesthesia and operative time, blood loss, and chest tube removal time. The results were found to be comparable between the two groups [36]. However, in a study by Iron et al., non-intubated surgeries were associated with shorter operative and hospital stays, reduced postoperative pain and oxygen requirements, and faster recovery [37]. In our study, operative time, hospital stay, and postoperative pain were found to be similar to intubated procedures. However, patients were protected from the adverse effects of general anesthesia exposure.

The utilization rate of VATS for lung diseases requiring biopsy has been reported to be 93–95% in various studies. These procedures are typically performed under general anesthesia, with intubation and single-lung mechanical ventilation [38,39]. Our study demonstrated that NI-UniVATS is as feasible as conventional VATS for lung diseases requiring biopsy, offering a viable alternative without the need for general anesthesia.

In thoracic surgeries, uncontrolled postoperative pain is commonly observed due to muscle incisions, rib retractions, and intercostal nerve injury [40,41]. Studies have shown that uniportal VATS (uniVATS) further reduces surgical trauma and postoperative pain [42,43]. In a study conducted by Zheng X et al., which included 68 patients, uniVATS and multiportal VATS were compared, and VAS scores at 24 and 72 h were significantly lower in the uniVATS group compared to the multiportal VATS group [44]. In our study, consistent with the literature, all operated patients underwent pain assessment at the first hour (VAS1), sixth hour (VAS2), and 24th hour (VAS3). Pain was moderate to severe at VAS1, mild to moderate with a significant reduction at VAS2, and very mild at VAS3, indicating a substantial decrease in pain levels over time.

Additionally, the volume of the center where these procedures are performed, as well as the collaboration and mutual trust between the anesthesiologist and the surgeon, will play a crucial role in achieving the desired outcomes [45]. This cooperation enables the procedure to be performed while avoiding intubation, thereby preventing the need for intensive care admission or potential extubation-related complications upon patient awakening.

Our study has several limitations. The small sample size, single-center design, and retrospective nature of the study are considered its primary weaknesses. However, despite these limitations, we believe that NI-UniVATS has the potential to become a routine clinical practice through multicenter, large-sample, and prospective studies. Further clinical research will strengthen the evidence regarding the safety and efficacy of this technique.

The long-term outcomes of NI-UniVATS will be a crucial indicator for its wider acceptance in clinical practice. Future prospective and long-term studies will provide a clearer understanding of its effectiveness, impact on patients’ quality of life, and complication rates. Our study contributes positively to the existing literature in this regard.

## 5. Conclusions

NI-UniVATS is a safe and feasible approach for both minor and major thoracic procedures. By eliminating the adverse effects and complications associated with general anesthesia, NI-UniVATS enables a safer surgical experience. The narrowing contraindication spectrum for NI-UniVATS clearly indicates that this technique can be safely utilized in almost all patient groups and has the potential for further development and broader clinical application.

## Figures and Tables

**Table 1:** Perioperative numerical data of the patients according to their clinical diagnoses.

	Pneumothorax	Pleural Effusion	Pulmonary Nodul	Mediastinal Mass	Pericardial Effusion	Number (n)
Age, (mean ± StD, min-max)	26.57 ± 8.62	60.60 ± 14.36	65.00 ± 7.51	46.33 ± 21.82	51.0	
Sex						
Male	19 (100)	12 (52.2)	3 (60.0)	2 (66.7)	1 (100)	37
Female	0 (0)	11 (47.8)	2 (40.0)	1 (33.3)	0 (0)	14
Location						
Right	8 (42.1)	16 (69.6)	3 (60.0)	1 (33.3)	0 (0)	28
Left	11 (57.9)	7 (30.4)	2 (40.0)	2 (66.7)	1 (100)	23
Length of stay in Hospital	3.84 ± 1.25	4.39 ± 1.87	3.80 ± 1.78	5.66 ± 3.51	3.0	
Operation Duration, min, (mean ± StD, min-max)	21.94 ± 6.81	21.17 ± 6.32	28.0 ± 7.44	33.33 ± 2.88	40.0	

**Table 2:** Preoperative comorbidities and intraoperative complications observed, classified according to patients’ clinical diagnoses.

	Pneumothorax	Pleural Effusion	Pulmonary Nodul	Mediastinal Mass	Pericardial Effusion	Number (n)
Comorbidity						
Heart failure	0 (0)	4 (33.3)	1 (50)	0 (0)	0 (0)	5
Chronic Obstructive Pulmonary Disease	1 (100)	2 (16.7)	1 (50)	0 (0)	0 (0)	4
Chronic Renal Failure	0 (0)	1 (8.3)	0 (0)	0 (0)	0 (0)	1
Diabetes Mellitus	0 (0)	3 (25)	0 (0)	0 (0)	0 (0)	3
Obesity	0 (0)	2 (16.7)	0 (0)	0 (0)	0 (0)	2
Surgery-impairing factors						
Panic Attack	1 (33.3)	0 (0)	0 (0)	0 (0)	0 (0)	1
Cough	2 (66.7)	0 (0)	1 (100)	0 (0)	0 (0)	3

**Table 3:** Postoperative pain scoring according to the clinical diagnoses of the patients.

	Pneumothorax	Pleural Effusion	Pulmonary Nodul	Mediastinal Mass	Pericardial Effusion
VAS1, (mean ± StD, min-max)	5.73 ± 1.28	6.0 ± 1.31	5.0 ± 1.0	6.66 ± 2.30	4.0
VAS2, (mean ± StD, min-max)	3.26 ± 0.45	3.39 ± 0.65	3.40 ± 1.14	3.33 ± 1.15	3.0
VAS3, (mean ± StD, min-max)	1.73 ± 0.45	1.65 ± 0.64	2.0 ± 0.70	2.0 ± 0.10	2.0

**Table 4:** Postoperative pathological diagnoses based on the surgical procedures performed and the collected specimens.

	Count (n)	Percent (%)
Operation		
Biopsy	27	52.9
Wedge Resection	22	43.1
Pericardial Fenestration	1	2
Foreign Body Removal	1	2
Pathological Diagnosis		
Bullae	19	37.3
Fibrinous Pleuoritis	10	19.6
Metastasis (Adeno ca)	7	13.7
Mesothelioma	6	11.8
Hamartoma	2	3.9
Lymphoma	2	3.9
Tuberculosis	3	5.9
Fibrous Tumor	1	2.0
Pericarditis	1	2.0

**Table 5:** Comparison of patients with pneumothorax and pleural effusion operated with NI-UniVATS.

	Pneumothorax n (%)	Pleural Effusion n (%)	*p*
Age, (mean ± StD, min-max)	26.25 ± 8.62	60.60 ± 14.36	<0.001
Sex			<0.001
Male	19 (100)	12 (52.2)	
Female	0 (0)	11 (47.8)	
Location			0.118
Right	8 (42.1)	16 (69.6)	
Left	11 (57.9)	7 (30.4)	
Operation			<0.001
Wedge Resection	18 (94.7)	0 (0)	
Biopsy	1 (5.3)	22 (95.7)	
Operation Duration, min, (mean ± StD, min-max)	21.91 ± 6.81	27.17 ± 6.32	0.005
Length of stay in Hospital	3.84 ± 1.25	4.39 ± 1.87	0.435
VAS1, (mean ± StD, min-max)	5.73 ± 1.28	6.0 ± 1.31	0.385
VAS2, (mean ± StD, min-max)	3.26 ± 0.45	3.39 ± 0.65	0.338
VAS3, (mean ± StD, min-max)	1.73 ± 0.45	1.65 ± 0.64	0.491

**Table 6:** Articles related to NI-VATS.

First Author	Years	Reference No.	Number of Patients	Study Design
Elkhouly et al.	2022	[10]	140	Retrospective
Palaniappa et al.	2023	[11]	54	Retrospective
Furak et al.	2020	[12]	160	Case series
Mineo et al.	2017	[13]	55	Non-randomized comparison
Starke et al.	2020	[14]	88	Case series
Li et al.	2020	[15]	57	Case series
AlGhmadi et al.	2018	[16]	31	Non-randomized comparison
Mugnaini et al.	2023	[17]	10	Retrospective
Farkas et al.	2023	[18]	32	Retrospective

## Data Availability

Datasets generated or analyzed in this study are accessible upon reasonable request from the corresponding author.

## Abbreviations

The following abbreviations are used in this manuscript:

NI-UniVATS	Non-intubated uniportal video-assisted thoracoscopic surgery
VATS	Video-assisted thoracoscopic surgery
VAS	Visual Analog Scale
ASA	American Society of Anesthesiologists
BMI	Body Mass Index
COPD	Chronic Obstructive Pulmonary Disease
SPSS	Statistical Package for the Social Sciences
